# Implementing evidence-based medicine practice in Indonesia: before, now, and the future

**DOI:** 10.1186/1687-9856-2013-S1-O4

**Published:** 2013-10-03

**Authors:** Sudigdo Sastroasmoro

**Affiliations:** 1 Department of Child Health/Center for Clinical Epidemiology and Evidence-based Medicine (CEEBM), Faculty of Medicine University of Indonesia – Cipto Mangunkusumo General Hospital, Jakarta, Indonesia

## 

Evidence-based medicine (EBM) is considered as one of the greatest inventions in modern medicine, advocating the use of the best available valid evidence, in line with clinical expertise and patients’ preference, to provide the best patient care. The philosophy probably has existed since the earliest age of medicine, but it was not until 1972 when Professor Archie Cochrane introduced the concept in one of his book, after which it was developed into a practical methodology by a group of scientists in the late 1980s and early 1990s. The work of EBM in Indonesia was first initiated in the early 2000s by conducting a series of intensive trainings at various medical teaching centres all over Indonesia. Most of the pioneers of EBM dissemination in Indonesia are paediatricians. Since then, teaching activities have been flourishing, leading to the formal incorporation of EBM into medical curricula in some medical schools. Collaborations with similar centres in Europe and Asia have a positive impact. Efforts are also being made in promoting the application of EBM principle in daily practice, such as in ward rounds, journal readings, and case discussions, both in undergraduate and postgraduate education settings. The term and practice of EBM have become very familiar for most health professionals in Indonesia, although occasionally it is misinterpreted in a narrow perspective, seeing evidence as the single factor determining health care. However, the implementation of EBM in clinical practice sometimes is hampered by the scarcity of relevant valid evidence like those happening in the field of paediatric endocrinology. This requires the transition of EBM practice in the future, in which health professionals do not merely act as evidence users but also play roles as evidence producers by conducting more patient-oriented research relevant to actual health problems. This is indeed very important, since most of the evidence available comes from studies in Western countries. Indonesian Clinical Epidemiology and Evidence-based Medicine (ICE-EBM) Network which currently has more than 30 member institutions has an important role in the acceleration of evidence-based practice in Indonesia.

